# Implications of Rho GTPase Signaling in Glioma Cell Invasion and Tumor Progression

**DOI:** 10.3389/fonc.2013.00241

**Published:** 2013-10-04

**Authors:** Shannon Patricia Fortin Ensign, Ian T. Mathews, Marc H. Symons, Michael E. Berens, Nhan L. Tran

**Affiliations:** ^1^Cancer and Cell Biology Division, Translational Genomics Research Institute, Phoenix, AZ, USA; ^2^Cancer Biology Graduate Interdisciplinary Program, University of Arizona, Tucson, AZ, USA; ^3^Center for Oncology and Cell Biology, The Feinstein Institute for Medical Research at North Shore-LIJ, Manhasset, NY, USA

**Keywords:** glioblastoma, invasion, survival, RhoGTPase, Rac1, Cdc42, RhoA, GEFs

## Abstract

Glioblastoma (GB) is the most malignant of primary adult brain tumors, characterized by a highly locally invasive cell population, as well as abundant proliferative cells, neoangiogenesis, and necrosis. Clinical intervention with chemotherapy or radiation may either promote or establish an environment for manifestation of invasive behavior. Understanding the molecular drivers of invasion in the context of glioma progression may be insightful in directing new treatments for patients with GB. Here, we review current knowledge on Rho family GTPases, their aberrant regulation in GB, and their effect on GB cell invasion and tumor progression. Rho GTPases are modulators of cell migration through effects on actin cytoskeleton rearrangement; in non-neoplastic tissue, expression and activation of Rho GTPases are normally under tight regulation. In GB, Rho GTPases are deregulated, often via hyperactivity or overexpression of their activators, Rho GEFs. Downstream effectors of Rho GTPases have been shown to promote invasiveness and, importantly, glioma cell survival. The study of aberrant Rho GTPase signaling in GB is thus an important investigation of cell invasion as well as treatment resistance and disease progression.

## Glioma Characterization

Gliomas comprise the most common group of primary brain tumors and are of neuroepithelial designation, named for a glial cell origin (1). These tumors include astrocytomas, oligodendrogliomas, and ependymomas, with infiltrating astrocytomas accounting for approximately 80% of adult primary brain tumors (2). Within each glial cell lineage, tumors are divided into grades based on their biologic behavior across one of four grades of increasing aggressiveness (grade I to grade IV); grading confers some prognostic guidance as well as informs treatment protocols. Clinically, glioblastoma (GB) (grade IV) represents the most malignant primary brain tumors, for which there is no cure (2).

Glioblastoma tumors arise in two scenarios: primary GB with new onset disease, or secondary GB with a previous history of lower grade astrocytoma. Gliomas rarely metastasize outside the CNS (2), but can be highly invasive within the brain parenchyma. Both lower grade as well as high-grade tumors display marked invasiveness, suggesting this malignant phenotype may be acquired early in tumorigenesis (3). Gliomas preferentially invade along white-matter tracts of the cerebrum, at times crossing the corpus callosum. Other patterns of cell spreading include perivascular growth, subpial spread, or perineuronal satellitosis (3).

The 5-year survival rate for GB is under 10% (4). Unfortunately, prognosis for GB patients has not significantly changed over the past several decades despite increased molecular understanding of the tumor. The problem of resistance to standard anti-proliferative treatment with concomitant radiotherapy and chemotherapy using the alkylating agent temozolomide (TMZ) is common, particularly manifest by the invasive cell population with subsequent tumor recurrence and death. The role of pro-invasive genes in GB progression remains understudied and the link between cell invasion and therapeutic insensitivity remains poorly characterized. Notably, treatments directed at impairing mediators of invasion have been shown to increase chemotherapeutic sensitivity (5–7).

Glioma cell invasion is promoted via the overexpression of receptors such as MET or EGFR, as well as downstream signaling through PI3K and MAPK pathways, and the Rho family of GTPases, among others (8, 9). This increased invasive potential arises from overexpression or mutation of drivers of cell motility, from interactions of glioma cells with pro-migratory signals within the brain tumor microenvironment, but also, notably, as a response to treatment (10). The Rho family of small GTPases, including primarily Rac1, Cdc42, and RhoG are key signaling mediators of GB cell invasion. Members of this signaling family as well as their regulators and effectors represent novel molecular targets against which therapeutic intervention could be aimed, and the implications for their signaling deregulation in gliomas is the subject of this review.

## Rho GTPase Function and Regulation

**Figure 1:**
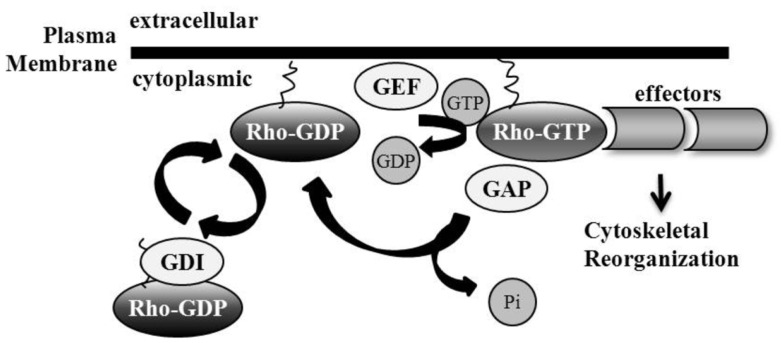
**Regulation of Rho GTPases**. Rho GTPase family members are active in their GTP-bound state, mediated by guanine nucleotide exchange factor (GEF) loading of GTP. GTPase-activating proteins (GAPs) catalyze intrinsic Rho GTPase GTP-to-GDP hydrolysis, including the removal of phosphate and the subsequent inactivation of the Rho GTPase. Guanine nucleotide dissociation inhibitors (GDIs) sequester Rho GTPases in their inactive GDP-bound conformation by shielding their hydrophobic tail. Membrane interaction and attachment is important for Rho GTPase activation and the promotion of subsequent downstream effector binding.

The Rho family of GTPases belongs to the Ras superfamily; they are monomeric low molecular weight proteins. There are 20 known mammalian Rho protein members, divided in part into subfamilies including Rho, Rac, Cdc42, Rnd, RhoD, RhoF, RhoH, and RhoBTB (11–16). Rho GTPases exist in either an inactive GDP-bound state, or an active GTP-bound state during which the GTPase can interact with downstream effectors. Switching between the inactive and active conformations is mediated by three classes of proteins: Guanine nucleotide exchange factors (GEFs), GTPase-activating proteins (GAPs), and Rho GDIs. GEFs are responsible for GTP loading. Stimulation of Rho GTPase intrinsic GTP-to-GDP hydrolysis by GAPs facilitates Rho GTPase inactivation (17). Rho GTPases undergo post-translational prenylation modifications, which are attached to the carboxyl-terminal cysteine residue of the protein, allowing for interactions with, and attachment at, phospholipid membranes where Rho GTPases are activated and interact with signaling complexes (18, 19). A third category of Rho GTPase family regulators are the guanine nucleotide dissociation inhibitors (Rho GDIs) which sequester Rho GTPases in their inactive conformation in the cytosol by shielding the hydrophobic tail (20). Rho GTPase activity can be regulated by nucleotide binding and subcellular localization (21). Recent studies have also shown Rho GTPase regulation via ubiquitin-proteasome degradation (22), as well as the importance of Rho GDIs in controlling the homeostasis of Rho proteins potentially by preventing protein degradation (23) (Figure 1).

RhoA, Rac, and Cdc42 are well-characterized members of the Rho family of GTPases and have been described as key regulators of cell migration (17). RhoA was first described to promote the assembly of contractile actomyosin filaments, or stress fibers, and Rac promoted the assembly of a peripheral actin meshwork, including lamellipodia (17, 24, 25). In addition to inducing peripheral actin rich microspikes, or filopodia, Cdc42 has been described to activate Rac, demonstrating the existence of cross-talk within the Rho family of GTPases (17, 26). Furthermore, RhoA and Rac1 have been shown to mutually inhibit each other in some studies, while other reports postulate a positive feedback (27). Another Rho GTPase family member, RhoG, has been shown to act upstream of Rac1 to stimulate cell movement, but also signals to promote the robust formation of Rac1-independent lamellipodia (28–31).

During the process of cell migration, actin-driven protrusions at the leading edge of cell motility are driven by Rac activation, whereas actomyosin contractility at the cell body and rear are coordinated by active Rho (32), with Cdc42 regulating cell polarity through integrating extracellular directional cues (33, 34). Rac and Cdc42 share an overlapping set of downstream effectors to enact cytoskeletal changes. Signaling via Pak and MAPK activation, or via PI5K, Formin, or IQGAP leads to actin reorganization downstream of either Rac or Cdc42. In addition, Cdc42 further signals via WASP and Rac further signals via WAVE to enact cytoskeletal rearrangement (35). RhoG, which has been described to signal both in concert with or in parallel to Rac1 and Cdc42 also shares downstream effectors IQGAP2, MLK3, and PLD1 with Rac1 and Cdc42, but confers independent signaling as well (31). RhoA signaling leads to phosphorylation of myosin light chain (MLC) and is conferred by several downstream effectors including the serine/threonine kinase p160 ROCK (36–38). ROCK activity leads to increased MLC phosphorylation via the inhibition of MLC phosphatase as well as direct MLC phosphorylation (36, 38, 39). ROCK, together with the RhoA effector mDia, coordinates stress fiber formation (38, 40).

While these signaling pathways are well-characterized, the understanding of Rho GTPase regulation of cell movement has become more complex. Tumor cell motility has been characterized to occur not only in a mesenchymal pattern with a spindle like shape and an obvious leading cell edge, but also in an amoeboid fashion with cycles of expansion and contraction of the cell body, potentially dependent upon the environment through which the cells move (41, 42). Moreover, biosensors capable of visualizing active Rho family members have implicated that both Rho and Rac can be active at leading edge protrusions (43). Thus the environmental regulation and spatiotemporal control of Rho GTPases are key factors in the regulation of cytoskeletal dynamics toward cell locomotion.

The activity of Rho GTPases is tightly regulated. Rho GEFs belong either to the Dbl or Dock families. Dbl proteins contain a Dbl homology (DH) domain responsible for catalyzing the exchange of GDP for GTP (12); DH domain binding to the Rho GTPase switch region induces conformational remodeling of the nucleotide-binding pocket (44). Pleckstrin homology (PH) domains are C-terminal adjacent associated regions to the DH domain that regulate GEF activity (45). PH domains bind phosphoinositides which facilitates plasma membrane localization, although it has also been suggested that other domains are necessary for directing subcellular localization (12). Furthermore, there is evidence that GEFs may be negatively regulated by sites in their N-terminus. The constitutive activation of many Rho GEFs occurs following removal of N-terminal sequences with *in vivo* protein expression (45); thus, the N-terminal region may act to auto-inhibit the DH domain, the control of which can be relieved by phosphorylation (12). Within the Dock family there are 11 members that are characterized by the presence of two conserved domains, termed Dock-homology region-1 and -2 (DHR1 and DHR2). For some Dock proteins, DHR2 has been shown to be sufficient for catalytic activity (12, 46, 47). The mechanisms of signal activation, including GEF localization and protein interaction, relief of auto-inhibition, and alteration of activity for Rho GEFs remain poorly characterized.

## Deregulation of RhoGTPases in Gliomas

Although Rac1-activating mutations are newly discovered and described via exome sequencing in melanoma tissue (48, 49), to date there are no reports of these mutations in other tumor types including GB. Increased activity of Rac1 has been reported in GB, and data supporting the role of additional Rho GTPases including Cdc42, RhoG, and RhoA in GB progression have been detailed as well, the findings of which are described below.

### Rac

The levels of Rac1 protein correlate with tumor grade in astrocytomas. In GB, Rac1 prominent plasma membrane staining is observed, indicating a potential hyper-activation status (50). Additionally, Rac1 promotes invasive glioma cell behavior (50, 51). While most data supports the role of Rac1 in GB progression, the Rac3 GTPase, which has high homology to Rac1, has also been described to play a role in GB cell invasion; the siRNA-mediated depletion of Rac3 led to strong inhibition of GB cell invasion *in vitro* (52).

Rac1 facilitation of glioma cell invasion occurs via signaling through several receptors and effectors. The tumor necrosis factor receptor superfamily (TNFRSF) contains two members known to utilize Rac1 in GB. Downstream of the fibroblast growth factor-inducible 14 receptor (Fn14), the Rac1 protein is important in promoting the TNF-like weak inducer of apoptosis (TWEAK) ligand-induced activation of the Akt and NF-κB-pathways, and Fn14 signaling through Rac1 promoted increased cell invasion and resistance to cytotoxic therapy-induced apoptosis (51, 53, 54). Additionally, Fn14-induced Rac1 activation is mediated by Cdc42. TWEAK-Fn14-induced Rac1 activation was dependent upon the presence of Cdc42 protein, while Rac1 depletion had no effect on TWEAK-induced Cdc42 activity (55). Moreover, TWEAK-Fn14 signaling has been demonstrated to induce Rac1 activation through TNF receptor associated factor 2 (TRAF2)-dependent activation of SGEF and RhoG (56). Another member of the TNFRSF family, TROY, is overexpressed in glioma cells and activates Rac1 signaling in a Pyk-2 dependent fashion, leading to enhanced GB cell motility (57).

Rac1 activation and promotion of cell migration and invasion in glioma is also seen downstream of signaling networks known to be utilized in neuronal signaling and development. The neuropeptide neurotensin induced activation of Rac1 in U373 GB cells which express three subtypes of neurotensin receptors; neurotensin enhances specifically the migration of cells cultured on laminin, with neurotensin-treated cells migrating more slowly when cultured on plastic (58). In addition, neuropilin-1 is a receptor for the semaphorin family of axon guidance molecules, and signaling through its ligand semaphorin3A promotes Rac1 activity and GB cell migration (59). Semaphorin 5A and its receptor plexin-B3, however, have been shown to significantly inhibit glioma cell migration and invasion, with concomitant inactivation of Rac1 through RhoGDIα and the promotion of glioma cell differentiation; semaphorin5A protein expression was significantly reduced in high-grade astrocytomas (60, 61). The axon guidance ligand Ephrin-B3 is overexpressed in GB cells and expression correlates with invading cells, with Ephrin-B3 co-localizing with Rac1 at areas of lamellipodia formation (62). The expression of Ephrin-B3 induced Rac1 activation in GB cells (62).

Rac1 has also been shown to regulate the formation of invadopodia, which are specialized formations of the plasma membrane that promote degradation of the extracellular matrix, an action critical in glioma cell invasion (52, 63). The depletion of either Rac1 or synaptojanin 2, a Rac1 effector with phosphatidylinositol phosphatase activity, decreases invadopodia formation, and glioma cell invasion (64). Synaptojanin 2 is also enriched in invadopodia (64). Additionally, Rac1 activation at the plasma membrane in glioma is regulated by Geranylgeranyltransferase I as well as RLIP76 modulation of ubiquitination (65, 66).

Rac1 signaling has also been reported to be activated downstream of several known receptor drivers of glioma malignancy. For example, Rac1 was activated subsequent to EGFRvIII-Src family kinase-dependent or protein kinase A-dependent phosphorylation of Dock180 (67, 68). Over half of “Classical” GB tumors, a genetically defined GB subtype, contain either point or *vIII EGFR* activating mutations (69), which results in a gain of function in-frame deletion of the extracellular portion of the receptor protein (70). Rac1 activation may be a key event in tumors of this subtype. Similarly, the PDGFRα receptor, which is amplified in a significant proportion of gliomas (71), induced Src-dependent Dock180 phosphorylation, with subsequent increased Rac1-GTP activity and GB cell growth and invasion (72). Lastly, Rac1 activation has been described downstream of several other Ras or Rho superfamily GTPases, including Rac1 activation subsequent to IQ-domain GTPase-activating protein 1 (IQGAP1)-dependent ADP-ribosylation factor 6 (ARF6) signaling (73). Rac1 activation downstream of additional Rho GTPase family members in GB is detailed below.

### Cdc42 and RhoG

There have been multiple described mechanisms of Rac1 activation in GB. Cdc42 and RhoG both share an overlapping set of activators and effectors with Rac1, and have been shown to function upstream of Rac1 in the regulation of several biological functions, including cell polarity and cell migration (29, 30, 35, 74–77). In GB, there is data to support the activity of Cdc42 and RhoG upstream of Rac1 activation; however the overall data on the status of these GTPases in GB is limited. The TWEAK-Fn14 ligand receptor axis-induced activation of Rac1 is one mechanism to enhance Rac1-GTP that is dependent upon a functional and activated Cdc42 protein; the depletion of Cdc42 abrogated glioma cell migration *in vitro* and invasion *ex vivo* (55). Cdc42 activation has also been demonstrated alongside Rac1 activation in GB cells downstream of PDGFRα association with SHP-2 non-receptor protein tyrosine phosphatase and Dynamin 2 (Dyn2) to promote glioma cell migration (78). The activation of Rac1 in neurotensin neuropeptide treated glioma cells was described in parallel with Cdc42 activity, although the dependence of one GTPase on the other for activation was not explored (58). In addition, biosensor studies have shown that both Cdc42 and Rac1 display high activity in the leading GB cells in the process of penetrating the brain parenchyma (79).

RhoG protein levels are elevated in GB (77), and RhoG has been reported to stimulate lamellipodia formation and confer downstream activation of Rac1 with a subsequent increase in cell migration (28–30, 77). Furthermore, TWEAK-Fn14 signaling in GB induces the rapid activation of RhoG dependent upon the receptor recruitment of TRAF2 and SGEF-mediated guanine nucleotide exchange, leading to increased levels of active Rac1 (56). RhoG can also activate Rac1 and Cdc42 in the regulation of neuronal process plasticity (80, 81), thus it remains a possibility that deregulated neuronal signaling and development pathways may also utilize RhoG to promote glioma malignancy. In addition, RhoG promoted caveolar endocytosis of growth factor receptors, including the regulation of EGF receptor internalization (82, 83). In GB, RhoG is activated downstream of EGF signaling and promotes GB cell migration (77); RhoG, therefore, may play a yet unidentified role in receptor trafficking within GB tumors.

There have also been several Rac1-independent functions of Cdc42 and RhoG described in other signaling systems (31, 84–86), although the data in GB is limited. Depletion of the Cdc42-specific GAP ARHGAP21 altered GB cell morphology and increased Cdc42 activity, FAK phosphorylation, MMP-2 production, and the rate of cell migration (87). Moreover, the Slit2 axon guidance molecule, which has been characterized to have lower expression in primary glioma specimens and invasive glioma cells relative to non-neoplastic brain, was described as part of the Slit2-Robo1 axis that inhibited glioma invasion through attenuation of Cdc42 activity (88). Evidence for RhoG regulation of GB cell invasion in a Rac1-independent fashion has been demonstrated downstream of HGF-induced invasion. RhoG depletion abolished HGF-induced invasion, but only partially inhibited HGF-stimulated Rac1 activity (77), suggesting RhoG also can utilize Rac1-independent mechanisms to promote HGF-induced cell motility.

### RhoA

To date, much of the data on RhoA function in glioma remains largely correlative. Decreased RhoA activity occurred in correlation with increased glioma cell migration (51, 89–91). Functional evidence for the role of RhoA has been demonstrated under the inhibition of the RhoA effector ROCK, which led to activation of Rac1 in glioma cells and promoted invasion. Of note, this inhibition led to the cellular morphological changes including an increase in the number and length of cell processes, increased membrane ruffling, and collapse of actin stress fibers (92). In addition, the inhibition of Rho/ROCK downstream of LPA induced glioma cell chemotaxis also led to cells that displayed long thin morphologies with extension of processes (93). RhoA activation in astrocytoma cells has been demonstrated to confer cell process retraction and rounding in the presence of decreased Rac1 activity (94). Also, pharmacologic inhibition of Ras in GB resulted in decreased Rac1 activity with coincident increased RhoA activation and stress fiber formation leading to rigid matrix attachment and cell immobility (95). Similarly, pharmacologic EGFR inhibition led to the Rho/ROCK-dependent formation of stress fibers with consequent decreased glioma cell invasion (96). Additionally, treatment of GB cells with the plant growth modulator Narciclasine led to increased ROCK activity and stress fiber formation, and resulted in increased survival of orthotopic xenograft-bearing mice (97). One recent report links RhoA inhibition with decreased mesenchymal type invasion, however the inhibitor utilized also targets RhoB and RhoC, thus excluding a definitive conclusion on the involvement of RhoA (98).

The RhoA and Rac1 pathways are increasingly associated with the promotion of divergent modes of cell migration in other systems; amoeboid type motility, characterized by cell rounding has been demonstrated to be regulated by the Rho/ROCK pathway, whereas prominent Rac signaling regulates mesenchymal type invasion with long morphological process extension (99). These modes of migration have been demonstrated to occur on differing substrate rigidity and size (42). In GB there is insufficient functional evidence for the role of RhoA in promoting amoeboid cell migration.

It also is interesting to note that substrate preference in the mode of GB cell invasion may be influenced by RhoA signaling. FRET studies have shown that while activated Rac1 and Cdc42 was high in rat GB cells invading brain parenchyma, RhoA activity was high in the perivascular region where there was coincident lower activity of Rac1 and Cdc42 (79). Furthermore, the downstream Rho effector ROCK signaling has been demonstrated to be an important modulator of substrate preference for GB cell route of invasion into brain parenchyma (100).

## Role of GEFs in Glioma

Of the more than 80 characterized Rho GEFs, the contribution of GEFs to glioma pathobiology may be in large part due to their expression patterns. Five of the GEFs described to promote GB cell motility, including Ect2, Vav3, Trio, SGEF, and SWAP-70, are overexpressed in GB versus non-neoplastic brain, and Dock180 expression is higher in the tumor rim than in the tumor core (50, 56, 76, 101).

### Ect2, Beta-PIX, SWAP-70, SGEF

It is likely that tumor cell invasion depends on the specific intracellular localization of Rho GEFs. Ect2, a GEF with RhoA, Cdc42, and possible Rac1 activity, was initially shown to regulate cytokinesis and actin cortex organization during mitosis (102, 103). While low-grade astrocytomas show predominantly nuclear Ect2 staining; GBs display prominent Ect2 staining in both the cytoplasm and nucleus (50, 104). In GB cells, Ect2 confers exchange for Cdc42, and subsequently Rac1, downstream of TWEAK-Fn14 (55). Moreover, Ect2 co-localizes with Rac1 and Cdc42 in the membrane ruffles of migratory astrocytoma cells, and inhibition of Ect2 led to decreased Rac1 and Cdc42 activity, with no change in Rho activity (104). Ect2 may additionally promote mesenchymal-amoeboid transition via interaction with the Rho-GAP RASAL2 (104). Overexpression of Ect2 *in vivo* promotes astrocyte migration in mice (55). ARHGEF7 (βPIX), a GEF that acts on both Rac1 and Cdc42, has additionally been studied in the context of cell migration (105). In GB, βPIX has been shown to be recruited to the leading edge of cell migration by the TIP-1 PDZ-scaffolding protein, where co-localization with Rac1 and Cdc42 promotes cell motility (106).

The Rho GEF SWAP-70 was discovered as a primarily nuclear protein with weak DNA-binding affinity in activated B-lymphocytes (107). Recently, it has been shown to localize at the leading edge of migrating glioma cells where it promoted membrane ruffling and migration and invasion of glioma cells, as well as the EGF-induced activation of Rac1 (101). In addition, the RhoG-specific exchange factor SGEF, which was initially characterized as an androgen-responsive gene in human prostate cells, has been shown to induce membrane ruffling and macropinocytosis in human fibroblasts (108, 109). SGEF localization in non-transformed human keratinocytes was demonstrated to be primarily nuclear, although HPV-mediated transformation was shown to be dependent upon SGEF and RhoG cytoplasmic activity, leading to increased membrane ruffling and cell invasion (110). In GB, SGEF is overexpressed relative to non-neoplastic brain tissue, and promotes the RhoG-mediated activation of Rac1 downstream of TWEAK-Fn14 signaling leading to increased cell migration and invasion (56). Thus, the specific localization of GEFs in brain tumors may promote cell invasion.

### Vav

The Vav family of Rho GEFs has been shown to promote glioma malignancy, however their isoform-specific roles vary among tumor grade and cell type context dependence. Vav1 and Vav3 have independently been shown to be overexpressed in patients with high-grade gliomas; Vav1, however, is overexpressed in peritumoral and perivascular, non-neoplastic astrocytes and thus its role may be related to cross-talk between the microenvironment and glioma cells. In contrast, Vav3 expression is localized to the glioma cells and Vav3 was shown to mediate exchange for Rac1 in the promotion of glioma cell migration (50, 111). Vav2 has been shown to exchange for Rac1 and promote chemotactic cell migration via the G protein-coupled P2Y(2) nucleotide receptor [P2Y(2)R] in low-grade astrocytomas, but has not been reported to play a role in high-grade GB (112). Thus the Vav family of Rho GEFs may promote malignancy across varying grades of brain tumors.

### Trio

Trio is defined by a unique structure containing two active GEF domains, one with Rac-specific activity and the second with Rho-specific activity, as well as a protein serine/threonine kinase (PSK) domain (113). Trio is overexpressed in high-grade gliomas and correlates with poor patient outcome; the depletion of Trio inhibits glioma cell invasion *ex vivo* (50, 55). Trio has been shown to confer guanine nucleotide exchange for Rac1 downstream of TWEAK-Fn14-induced Cdc42 activity, with subsequent promotion of cell migration (55). The role of Trio exchange for RhoA has been unexplored in GB, however given that Rac1 and RhoA may regulate divergent modes of invasion, it remains possible that Trio plays a role in the regulation of mesenchymal-amoeboid shifts in the migration of GB cells.

### Dock

Within the Dock180 superfamily of proteins, Dock180 and Dock9 have been characterized in glioma. The Dock180 GEF signals as a bipartite GEF for Rac1 in connection with the PH domain of ELMO1, and expression of ELMO1 is elevated in a subset of GB (47, 114). Furthermore, the exogenous expression of ELMO1 and Dock180 in glioma cells enhances their migratory and invasive capacities *in vitro* and in brain tissue (76). In addition, the phosphorylation of Dock180 at tyrosine 1811 has been demonstrated to occur downstream of PDGFRα in glioma, promoting Rac1 activation and cell invasion, migration, and survival (72).

Dock9, also known as Zizimin1, is a Cdc42-specific Dock family GEF. Dock9 interacts with the plasma membrane via its N-terminal PH domain; the N-terminal region also serves to auto-inhibit the catalytic function of Dock9 (115, 116). Dock9 was initially characterized for its role in dendrite formation in rat hippocampal neurons (117), but has recently been shown to be important for GB cell invasion in the rat. GB cells with Zizimin1 knockdown displayed lower Rac1 and Cdc42 activity (79).

Roles for additional GEFs have yet to be defined in glioma, but further characterization of GEF localization and activation mechanisms may prove useful in understanding GB invasion.

## Glioma Pro-Invasive Pathways Promote Tumor Survival

Cancer invasion and drug-resistance are increasingly being recognized as interconnected processes sharing overlapping pathways that together promote disease progression and therapy failure (7). In GB, the tumor response to external stresses, including from the tumor microenvironment or from chemotherapeutic or radiation treatment, involves coordinated pro-survival and pro-invasion signaling. The GB microenvironment is comprised of areas of hypoxia notably including pseudopalisading necrosis, one of the histological hallmarks of GB, which has been defined to consist of tumor cells actively invading away from a hypoxic core of tissue following a vaso-occlusive event. These hypoxia-induced migratory cells have increased tumor cell glycolysis and secrete pro-angiogenic factors (118–120), and increased microvessel density in astroglial brain tumors is a prognostic indicator of poor patient survival (121). Thus, tumor hypoxia in GB promotes both cell invasion into normal brain tissue and supports angiogenesis for tumor survival and growth. Glioma cells with the increased capacity for migration have a decreased expression of pro-apoptotic genes and are less sensitive to cytotoxic therapy-induced apoptosis (51, 53, 122–124). Several of the Rho GTPases and Rho GEFs shown to promote GB cell invasion have also been correlated to poor patient prognosis among GB tissue specimens (50, 56, 72, 101), and signaling through Rho GTPases is part of the coordinated survival response following treatment in GB.

For example, chemotherapeutic resistance in glioma cells has been shown to be promoted through Rac1-dependent Akt2 activity working upstream of the BCL2 family to promote cell survival (54). Activation of Akt2 led to increased MMP-9 expression and increased glioma cell migration and invasion (125). The inhibition of NF-κB has been shown to promote increased glioma cell death, which was synergistic under the combined treatment with TMZ, and led to decreased migration and invasion with decreased expression of invasion-related genes (126). Furthermore, overexpression of the pro-invasive TNFRSF member TROY increased glioma cell resistance to irradiation or TMZ treatment dependent upon Akt and NF-κB activity, and depletion of TROY in orthotopically implanted primary GB xenografts led to increased survival of mice (127). In addition, sub-optimal dosing of TMZ cooperatively reduces cell growth in EGFR inhibited glioma cells with concomitant Rho/ROCK-dependent inhibition of glioma cell invasion (96). Furthermore, glioma stem cells are increasingly being recognized as TMZ resistant, both through intrinsic resistance mechanisms and via interaction with the brain parenchyma toward the promotion of extrinsic resistance (128). Rac1 has been shown to be important for the maintenance of stemness and tumorigenicity in human glioma, whereby depletion of Rac1 suppressed glioma stem-like cell migration, invasion, and malignant transformation, while conferring enhanced radiation sensitivity to these glioma stem-like cells (129).

**Figure 2:**
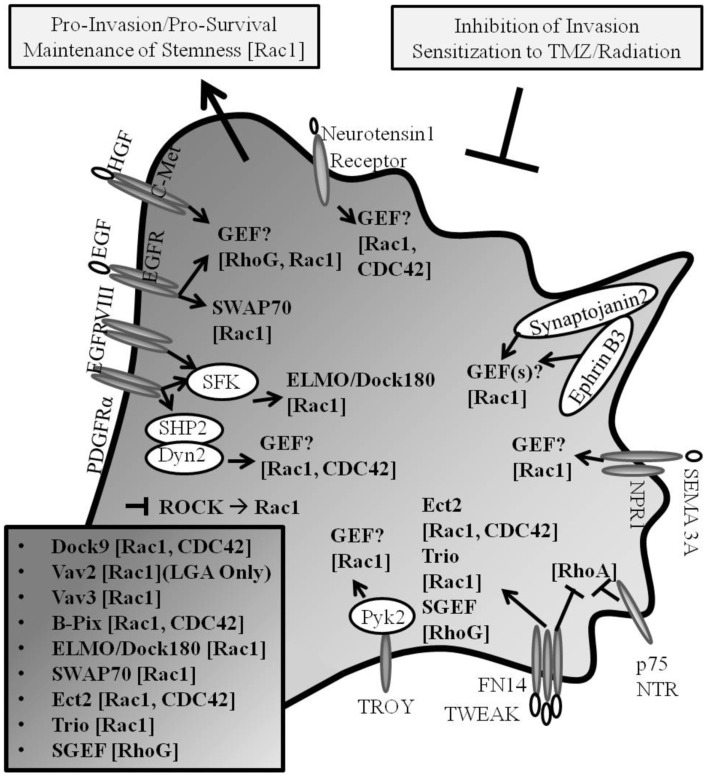
**Rho GEF and Rho GTPase signaling in GB**. Pro-invasive Rho GEF family members Dock9, Vav2, Vav3, β-Pix, ELMO/Dock180, SWAP-70, Ect2, Trio, and SGEF confer guanine nucleotide exchange for Rho GTPase family members Rac1, Cdc42, and RhoG in glioma progression (Known GEFs in glioma are summarized in insert with corresponding GTPases shown in brackets; LGA, lower grade astrocytoma only). Cancer invasion and drug-resistance share overlapping signaling pathways, including the signaling through Rho GTPases in glioma. The inhibition of mediators of invasion confers increased susceptibility to chemotherapeutic- and radiation-induced cell death.

The irradiation of primary GB cells has been demonstrated to enhance cell invasion (10). TRAF2, which has been shown to be recruited upstream of SGEF-RhoG-Rac1 pro-invasive signaling in the TWEAK-Fn14 axis (56), when depleted in GB has also been shown to inhibit growth and confer radio-sensitization to tumor cells (130). Additional reports have characterized that signaling through TRAF2 promotes not only NF-κB activity, but also JNK/SAPK activity, inflammation, and cell migration and chemo- and radio-resistance of cancer cells (131–137). In GB, JNK is activated downstream of Rac1, whereby activated JNK translocated to paxillin-containing focal complexes leading to paxillin phosphorylation at a site known to regulate cell migration (138). Moreover, it has been suggested that Rac may promote radiation therapy-induced increased glioma cell invasion in concert with activated p38 and JNK signaling (139). In addition, the pharmacologic inhibition of auto-phosphorylation of focal adhesion kinase in GB was shown to inhibit cell invasion and increase cell apoptosis, an effect which was synergistic when treated with TMZ *in vivo* (140). Taken together, Rho GTPases and Rho GEFs are implicated in the regulation of invasion and survival signaling in GB (Figure 2).

## Conclusion

Deregulated pathway signaling in GB tumors occurs within multiple processes including proliferation, metabolism, gliomagenesis, angiogenesis, survival, and invasion. Patient mortality is ultimately due to tumor spread and growth burden, thus the identification of key drivers of cell invasion can inform future targeted therapy development for use in clinical trials, with the intent to sensitize cells to combinatorial therapy with chemotherapeutic and radiologic interventions.

The acquisition of cell motility is complex and influenced by various intracellular and extracellular signaling events. The Rho family of GTPases are well-defined regulators of actin cytoskeletal dynamics (17, 24, 37), and have been characterized to contribute to most steps of cancer initiation and progression (141). Suppression of Rac activity selectively induces apoptosis in glioma cells but not in normal human astrocytes (142), thus proffering a rationale for the therapeutic inhibition of pro-migratory signaling pathways including those promoting Rac activation as an effective clinical option for GB. Increased support for the utility of Rho GTPase pathway inhibitors in treating cancer has led to the recent development of several strategies to identify drugs that will act against Rho GTPases, or their regulation by GEFs (143–151). These studies highlight the potential use of GEF inhibition and support both the rationale for and the feasibility of future GEF inhibitors for clinical use.

## Conflict of Interest Statement

The authors declare that the research was conducted in the absence of any commercial or financial relationships that could be construed as a potential conflict of interest.

## Abbreviations

GAP, GTPase-activating protein; GB, glioblastoma; GDI, guanine nucleotide dissociation inhibitor; GEF, guanine nucleotide exchange factor.
